# Cross-Species Transcriptomic Integration Identifies a Conserved Molecular Core and Regulatory ceRNA Network in Duchenne Muscular Dystrophy

**DOI:** 10.3390/genes17091017

**Published:** 2026-08-27

**Authors:** Abdolvahab Ebrahimpour Gorji, Tomasz Sadkowski

**Affiliations:** Department of Physiological Sciences, Institute of Veterinary Medicine, Warsaw University of Life Sciences, 02-776 Warsaw, Poland; abdolvahab_ebrahimpourgorji1@sggw.edu.pl

**Keywords:** Duchenne muscular dystrophy, RNA-seq, cross-species analysis, orthologs, circRNA, miRNA, ceRNA network, skeletal muscle

## Abstract

Background: Duchenne muscular dystrophy (DMD) is a monogenic disorder caused by dystrophin deficiency, but its downstream molecular consequences involve complex and interconnected regulatory networks. Methods: In this study, we performed a cross-species integrative transcriptomic analysis of skeletal muscle RNA-seq datasets from humans, mice, and rats to identify conserved molecular signatures of DMD. Results: Differential expression analysis identified 1367 shared differentially expressed genes (DEGs) across the three datasets, of which 62 showed concordant directionality in all species. After ortholog filtering, which excluded five genes lacking confirmed one-to-one orthologs across all three species, 57 conserved genes were retained as the conserved DMD-associated gene (DAG) set. Functional enrichment (GO/KEGG) was performed on the cross-species shared DEG set, and the 57 conserved genes were subsequently mapped onto these enrichment outputs. The DAG-containing categories included focal adhesion, apoptosis, and calcium signaling, which remained significant after Benjamini–Hochberg correction, together with nominally enriched Rap1 signaling. Integration of circRNA, miRNA, and mRNA data further identified a candidate, computationally inferred hub-dominated ceRNA network involving DMD, RYR3, KLF4, ID1, TIMP4, and CASP7. Conclusions: Together, these findings support a model in which conserved core molecular programs are superimposed on species- and context-dependent transcriptional responses, prioritizing the conserved genes and candidate circRNA-related network components for future mechanistic and translational studies.

## 1. Introduction

Duchenne muscular dystrophy (DMD) is one of the most common inherited neuromuscular disorders, affecting approximately 1 in 3500 live male births worldwide [1]. DMD is caused by pathogenic variants in the dystrophin gene (*DMD*), which encodes dystrophin, a critical structural protein in muscle fibers. Loss of dystrophin leads to progressive muscle degeneration, loss of ambulation, respiratory insufficiency, and cardiomyopathy, which often becomes life-limiting in early adulthood [2].

Although much attention has focused on clarifying the function of the *DMD* gene in DMD, it is now recognized that the disease, while monogenic, reflects systems-level disturbances driven by interactions among multiple genes and pathways downstream of the dystrophin mutation. It has been established that DMD adversely affects multiple molecular processes involved in muscle contraction, the arrangement and organization of the extracellular matrix (ECM), inflammation, and others [3]. Thus, several questions remain regarding the molecular basis of DMD and its potential therapeutic targets.

One possible strategy to reconcile the discrepancies in the current literature is centered on the concept of hub genes—genes that are highly connected in PPI networks and essential to disease-associated molecular events. These hub genes may serve as potential biomarkers or therapeutic targets due to their influence on multiple regulatory pathways within a cell [4].

In DMD, several hub genes play critical roles in the disease pathogenesis. Hub genes are central to gene networks and are often involved in key biological processes. Recent studies have identified several hub genes associated with DMD, including *SERPING1, F13A1*, *C1S*, *C1R*, *HLA-DPA1*, and others like *CD44*, *ECT2*, *TYMS*, *MAGEL2*, *HLA-DMA*, *SERPINH1*, and *TNNT2* [5,6]. These hub genes are primarily involved in immune response regulation, inflammation, and muscle regeneration. For instance, *SERPING1* and *F13A1* are linked to immune effector processes, which may contribute to the inflammatory environment observed in DMD [5]. Additionally, genes like *DMD* and *TTN* are crucial for muscle function and structural integrity, and their dysregulation can lead to muscle degeneration [6].

Moreover, identifying these hub genes through bioinformatics approaches, such as weighted gene co-expression network analysis, provides insights into potential therapeutic targets and biomarkers for this disease [7]. The transforming growth factor-β (TGF-β) signaling pathway has been characterized as a significant modifier of gene expression in muscular dystrophy. It is also enhanced through a destabilized plasma membrane and changes in the composition of the ECM of dystrophic muscle [8]. As such, characterizing the behavior of these hub genes will provide researchers with a better understanding of the broader molecular alterations in DMD, ultimately leading to the discovery of novel therapies and biomarkers for the disease.

**Figure 1:**
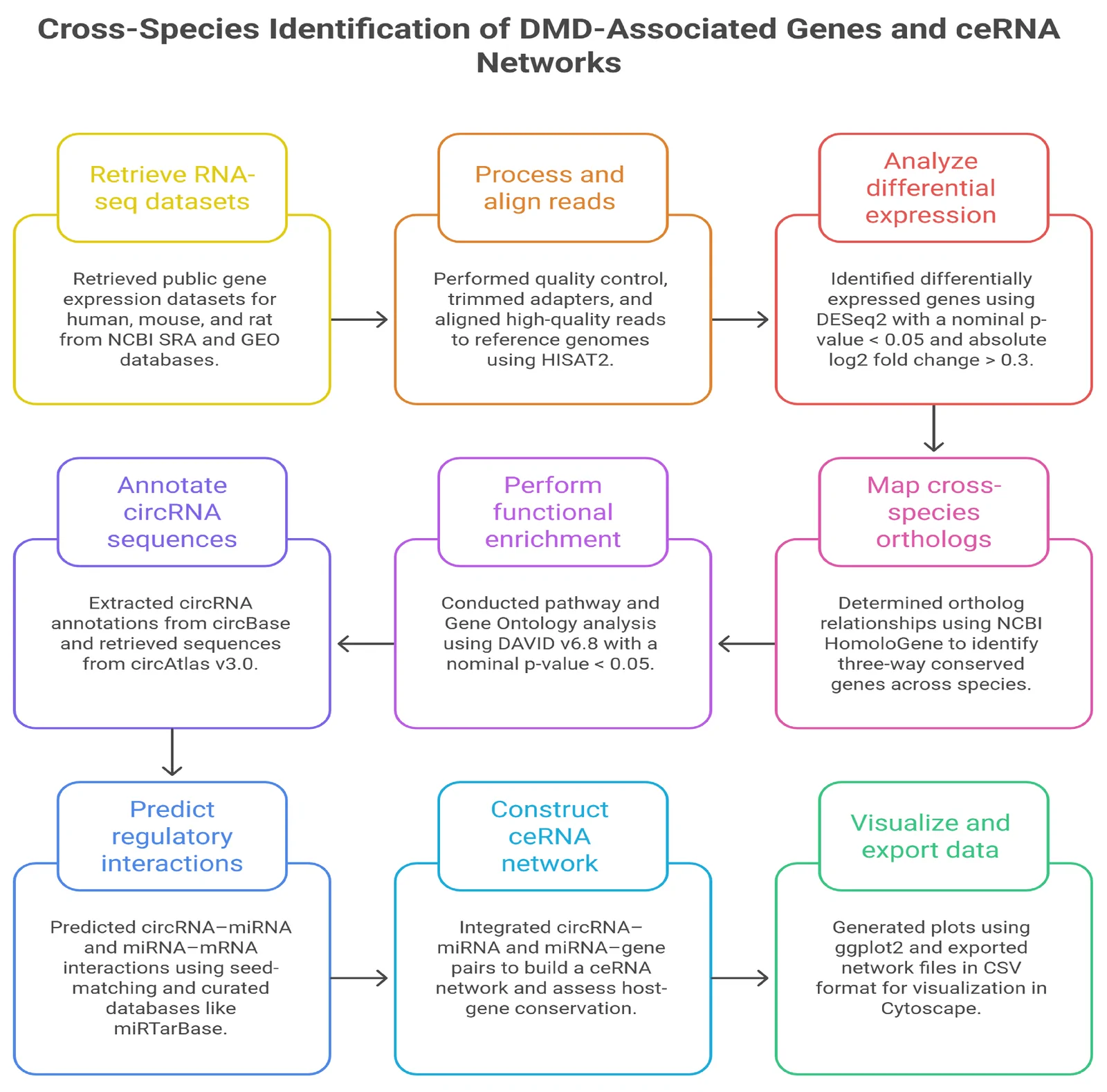
Overview of the analytical workflow: RNA-seq acquisition and alignment, differential expression analysis, cross-species ortholog mapping, functional enrichment, circRNA annotation, and ceRNA network construction and visualization.

In the present study, we used a cross-species bioinformatic analysis of RNA-seq datasets from humans, mice, and rats to identify molecular pathways associated with DMD. Expression data were obtained from publicly available RNA-seq datasets deposited in the National Center for Biotechnology Information (NCBI) Sequence Read Archive (SRA). We compared gene expression profiles from DMD patients and dystrophin-deficient animal models with those from healthy controls to identify recurrent differentially expressed genes (DEGs) shared across species and experimental contexts (Figure 1). This cross-species approach enhances generalizability and facilitates the identification of recurrent disease-associated pathways that are amenable to therapeutic targeting.

## 2. Materials and Methods

### 2.1. Data Collection and Samples

We used three species (human, mouse, and rat) for in vivo analysis to identify DMD-associated genes and pathways. Public gene expression datasets were retrieved from the NCBI SRA and GEO databases. Human (*tibialis anterior*): BioProject PRJNA976807 submitted by University of California, Los Angeles; eight DMD samples (SRR25143906–SRR25143913; *n* = 8) and five healthy controls (SRR24750875, SRR24750891, SRR24750893, SRR24750895, SRR24750902; *n* = 5). Mouse (*gastrocnemius*): BioProject PRJNA1009848; C57BL/10ScSn-Dmd*^mdx^* (SRR25765411, SRR25765412, SRR25765413, SRR25765414, SRR25765418; *n* = 5) and BL10 healthy control mice (SRR25765415–SRR25765417, SRR25765419, SRR25765420; *n* = 5) [9]. Rat (*biceps femoris*): RNA-seq from *DMD^mdx^* rats and wild-type (WT) controls (*n* = 4 per group), deposited under BioProject PRJNA1191685 (NCBI SRA) [10] (Table 1, Supplementary Table S1A–C).

### 2.2. Preparation of Data and Analysis of Gene Expression

Quality control of raw RNA-seq reads involved adapter trimming and removal of low-quality reads and ambiguous bases. The retained high-quality reads were then used for downstream analyses. We assessed read quality with FastQC v0.12.1 (http://www.bioinformatics.babraham.ac.uk/projects/fastqc/; accessed on 20 October 2025). Following quality control, RNA-seq reads were trimmed using Trimmomatic v0.39 [11] and filtered as described above. They were then aligned to the corresponding reference genomes using HISAT2 v2.2.1 [12]: human (GRCh38), mouse (GRCm38), and rat (Rnor_6.0). Species-specific genome indices and default alignment parameters were applied unless otherwise noted. Gene-level read counts were obtained with FeatureCounts v2.0.6, assigning reads to exonic features based on the appropriate GTF annotation for each species. The resulting sample-by-gene count matrix was used for differential expression analysis.

### 2.3. Differential Gene Expression Analysis

Differential gene expression analysis was performed in R version 4.5.2 using the DESeq2 v1.46.0 package [13]. For differential expression analysis, genes with a nominal *p*-value < 0.05 and an absolute log2 fold change > 0.3 were retained for downstream analyses. Because this study was designed as an exploratory cross-species integration, we applied a permissive statistical threshold to preserve moderate but potentially recurrent biological signals. To reduce false-positive interpretation, we subsequently applied additional biological filters, including cross-dataset gene overlap, ortholog conservation, functional enrichment, and ceRNA network integration. Accordingly, the results should be interpreted as hypothesis-generating rather than strictly confirmatory. As a sensitivity analysis, DEGs were additionally identified using Benjamini–Hochberg false discovery rate (FDR)-adjusted *p*-values (padj < 0.05), and cross-species overlap was re-examined under this stricter criterion (Supplementary Table S2). All genes retained under FDR correction were subsequently carried through the same ortholog-mapping step applied to the primary analysis. The number of FDR-supported genes remaining after ortholog filtering is reported in Section 3.1 and Supplementary Table S2. We generated volcano plots in R using the ggplot2 package to investigate DEGs in each species, displaying genes by log2 fold change. Only genes exceeding the predefined *p*-value and fold-change thresholds were marked in color.

### 2.4. Cross-Species Identification of DMD-Associated Genes and Ortholog Mapping

DEGs identified in human, mouse, and rat datasets were compared across species after ortholog mapping. Ortholog relationships were determined using NCBI HomoloGene for *Homo sapiens* (9,606), *Mus musculus* (10,090), and *Rattus norvegicus* (10,116). Genes with confirmed one-to-one orthologs in all three species were classified as three-way conserved, whereas genes lacking orthologs in one or both non-human species were recorded separately. A manually curated ortholog table was also prepared for established DMD-related genes to verify the automated assignments. Because the human, mouse, and rat datasets originate from independent studies with distinct sequencing platforms, reference genome builds, and experimental designs, direct joint normalization or batch-correction across species was not performed, as such approaches assume comparable technical and biological baselines that do not hold across species-level datasets. Instead, differential expression was estimated independently within each species-specific dataset, and cross-species conservation was assessed by ortholog overlap between independently derived DEG sets. This overlap-based strategy avoids the need to directly reconcile cross-species batch and platform effects and is consistent with standard approaches in comparative cross-species transcriptomics, in which the recurrence of independently derived signals across systems is used as evidence of biological conservation.

### 2.5. Pathway Analysis and Gene Ontology

DEG-based functional enrichment analysis for KEGG pathways and Gene Ontology (GO) terms was performed using the Database for Annotation, Visualization, and Integrated Discovery (DAVID) v6.8 (https://david.ncifcrf.gov/; accessed on 25 October 2025). Terms and pathways with a nominal *p*-value < 0.05 were reported as exploratory enrichment results. Where available, DAVID Benjamini–Hochberg FDR-adjusted values were also recorded to distinguish nominally enriched terms from terms supported after multiple-testing correction. In addition, the final ortholog-filtered DMD-associated genes (DAGs) were mapped to the GO and KEGG enrichment outputs to enable identification of DAG-associated terms and pathways and comparison of nominal and FDR-adjusted enrichment statistics. All reported nominal and FDR-adjusted enrichment statistics refer to the cross-species shared DEG set, with the 57 ortholog-filtered DAGs reported as mapped members of these DEG-derived terms and pathways Results were visualized with SRplot [14].

### 2.6. circRNA Annotation and Sequence Retrieval

Human circRNA annotations were obtained from circBase (hg19 build) (https://www.circbase.org/; accessed on 15 December 2025) [15]. CircRNAs located on standard chromosomes (chr1–22, X, and Y) were retained, and entries whose host genes matched DMD-associated genes were extracted. The number of circRNAs associated with each host gene was then summarized to identify circRNA-enriched loci. Spliced circRNA sequences were retrieved from circAtlas v3.0 (https://ngdc.cncb.ac.cn/circatlas/; accessed on 15 December 2025). Entries annotated as unknown or shorter than 30 nucleotides were excluded. circAtlas identifiers were mapped to the DMD-associated genes using the embedded host gene symbol. Where multiple circAtlas entries corresponded to a single circBase circRNA, the sequence with the closest reported spliced length was selected.

### 2.7. Prediction of circRNA–miRNA Interactions and miRNA–Target Pairs

Because circRNA and miRNA expressions were not directly quantified in the analyzed RNA-seq datasets, all circRNA–miRNA and miRNA–mRNA interactions described below were derived computationally from sequence-based seed-matching predictions and curated interaction databases, rather than from measured expression correlation. Accordingly, these interactions represent candidate regulatory relationships rather than experimentally confirmed regulatory events. Mature human miRNA sequences were downloaded from miRBase v22.1 (https://www.mirbase.org/; accessed on 15 December 2025), and only hsa- entries were retained. For each miRNA, the canonical 7-mer seed region (nucleotides 2–8) was extracted. miRNAs containing ambiguous bases within the seed region were removed. Reverse-complement seed motifs were generated and used to scan circRNA sequences for exact matches using gregexpr() in R v4.5. For each circRNA–miRNA pair, the number of matched seed sites was recorded as the number of binding sites. Interactions with at least two binding sites were considered stronger candidate sponge interactions. Database-derived miRNA–target interactions involving the DMD-associated genes were retrieved using the multiMiR Bioconductor package (v1.20.0) [16] with the parameters org = “hsa”, table = “validated”, and summary = TRUE. The analysis incorporated records from miRTarBase [17], TarBase [18], and miRecords [19]. Queries were performed in batches of 10 genes with a 3 s pause between requests.

### 2.8. Candidate ceRNA Network Construction and Host-Gene Conservation Analysis

A ceRNA network was constructed by integrating predicted circRNA–miRNA interactions with multiMiR-derived miRNA–gene pairs. For each miRNA targeting one or more of the DMD-associated genes, circRNAs containing matching seed-binding sites were considered putative upstream sponges. The final network comprised three node classes—circRNAs, miRNAs, and DMD-associated genes—and two edge classes: circRNA–miRNA and miRNA–gene. To assess conservation, the host gene of each circRNA in the network was cross-referenced with the ortholog table. CircRNAs derived from genes conserved in humans, mice, and rats were designated as candidate sponges originating from three-species-conserved host genes. As no expression correlation analysis was performed among circRNAs, miRNAs, and target mRNAs, all network edges represent predicted, sequence- or database-derived associations and should be interpreted as hypotheses for future experimental testing rather than as validated regulatory interactions.

### 2.9. Statistical Analysis, Visualization, and Network Export

All analyses were performed in R v4.5 using Biostrings v2.72 [20], multiMiR v1.20 [16], dplyr v1.1 [21], and ggplot2 v3.5 [22]. Summary statistics included the number of binding sites per circRNA–miRNA pair, the number of miRNAs associated with each circRNA, and the number of DMD-associated genes linked to each circRNA through shared miRNAs. The association between circRNA length and miRNA-binding partner number was assessed by linear regression. Plots, including bar charts, heatmaps, histograms, and scatter plots, were generated in ggplot2 at 300 dpi. Network files were exported in CSV format for visualization in Cytoscape v3.10. Node tables included node type and conservation status, whereas edge tables included interaction type and binding-site count.

## 3. Results

### 3.1. Gene Expression Analysis and Identification of Overlapping Genes

**Figure 2:**
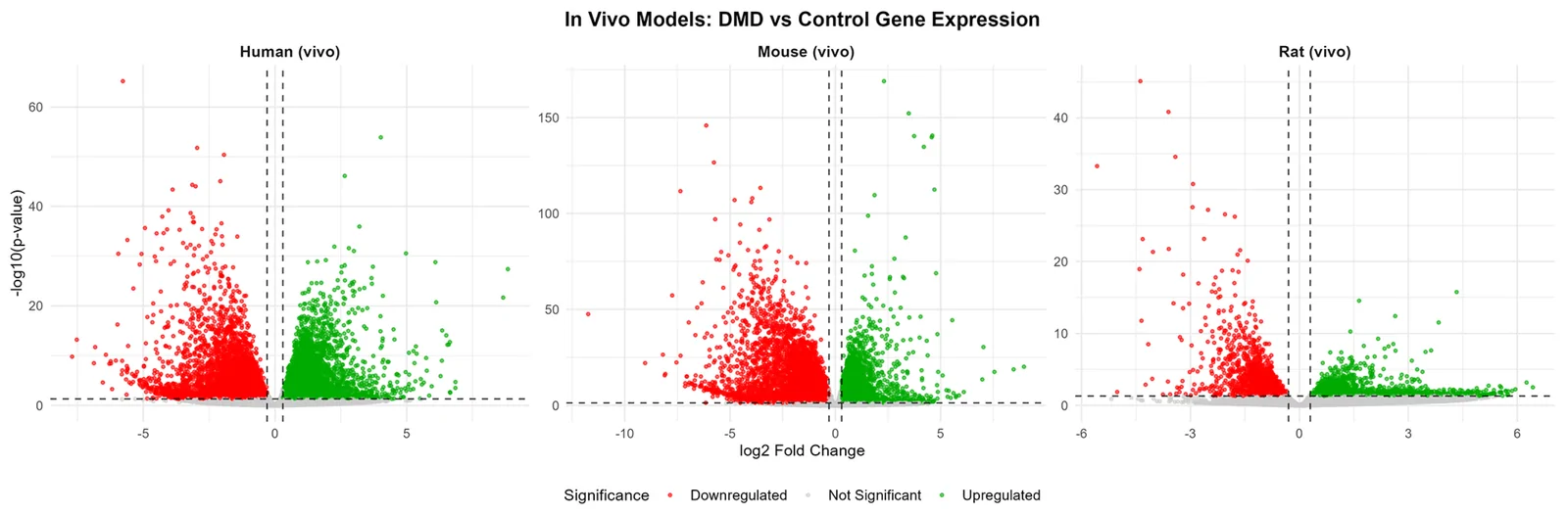
Volcano plots of differentially expressed genes in human, mouse, and rat skeletal muscle datasets. Each point represents a gene; genes upregulated in DMD are shown in green and genes downregulated in DMD are shown in red. The x-axis indicates log2 fold change (DMD vs. control), and the y-axis indicates −log10(*p*-value).

Differential expression analysis identified numerous genes that were significantly dysregulated in DMD versus control skeletal muscle samples across the human, mouse, and rat datasets. In this study, genes with *p* < 0.05 and |log2FC| > 0.3 were considered significant. In humans, 10,345 significant genes were identified (4994 downregulated and 5351 upregulated); in mice, 10,176 significant genes (5971 downregulated and 4205 upregulated), and in rats, 3785 significant genes (1814 downregulated and 1971 upregulated). A volcano plot for each species illustrates the distribution of significant DEGs, with upregulated and downregulated genes highlighted (Figure 2, Supplementary Table S1A–C). To assess the robustness of the conserved signature to the significance threshold used, we repeated the cross-species overlap analysis using FDR-adjusted *p*-values (padj < 0.05). This more stringent criterion markedly reduced the number of overlapping genes at each pairwise comparison (human–mouse: 146 upregulated, 58 downregulated; mouse–rat: 5 upregulated, 12 downregulated; rat–human: 6 upregulated, 22 downregulated) and reduced the three-species concordant overlap from 62 to 7 genes. All seven FDR-supported genes were included in the final ortholog-filtered DAG set: *CCBE1* and *RFX2* were upregulated, whereas *CASKIN2*, *DMD*, *ID1*, *KLF2*, and *KLF4* were downregulated (Supplementary Table S2). This ~89% reduction indicates that strict FDR correction substantially reduces the cross-species signal available for full downstream functional enrichment and ceRNA network analyses. We therefore retained the nominal *p*-value threshold as the primary exploratory screening criterion and used multi-layer biological filtering, including cross-dataset overlap, ortholog conservation, functional enrichment, and ceRNA integration, to prioritize a conserved DMD-associated signature.

**Figure 3:**
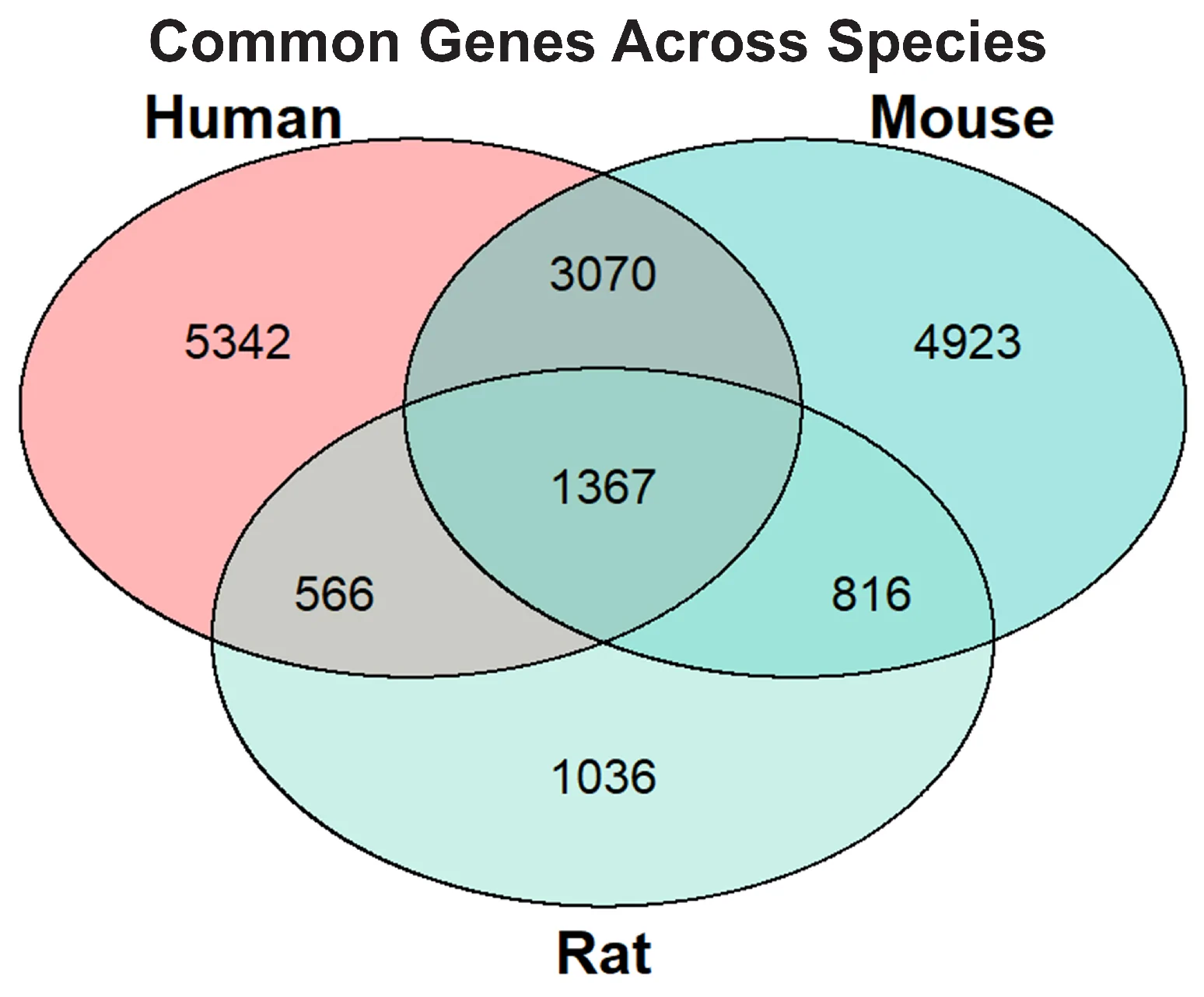
Venn diagram showing overlap of differentially expressed genes (DEGs) across skeletal muscle tissue samples.

**Figure 4:**
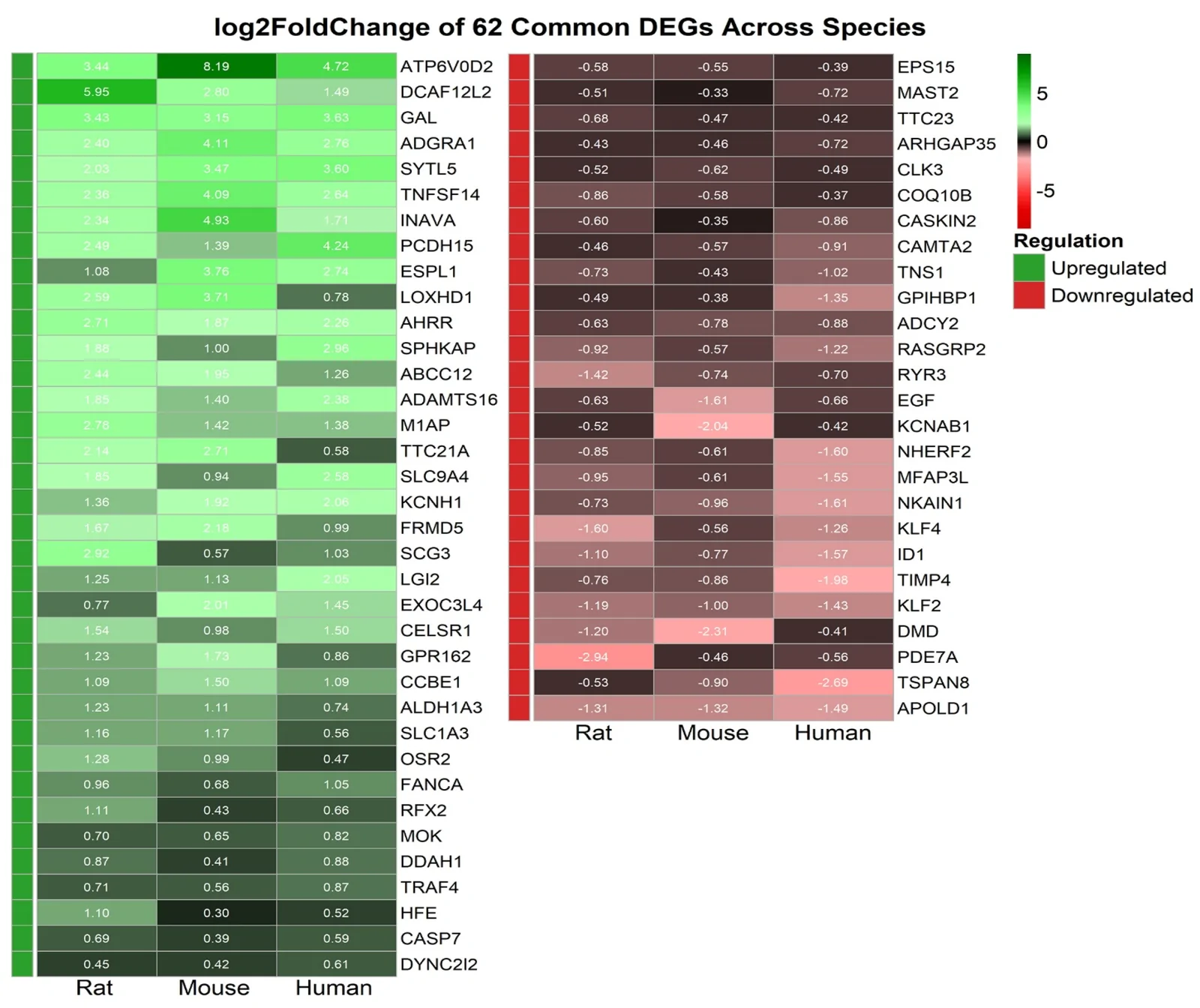
Heatmap of the 62 overlapping genes showing concordant expression direction across the human, mouse, and rat datasets.

Comparative analysis identified 1,367 DEGs shared across the three species, of which 62 showed concordant directionality in humans, mice, and rats. After ortholog filtering, 57 conserved DMD-associated genes (DAGs) were retained for downstream Gene Ontology (GO), pathway, and network analyses (Figure 3 and Figure 4).

### 3.2. Ortholog Conservation of the 62 Concordantly Dysregulated Genes

Cross-species ortholog mapping using NCBI HomoloGene identified one-to-one orthologs for the majority of the 62 concordantly dysregulated genes. Of the 62 genes, 57 (87.1%) had confirmed orthologs in both *M. musculus* and *R. norvegicus*, indicating strong gene-level conservation across all three mammalian species. A subset of 5 genes (*EXOC3L4*, *ABCC12*, *M1AP*, *DCAF12L2*, and *INAVA)* lacked high-conservation-confidence orthologs in one or both non-human species, suggesting these may represent lineage-specific or rapidly evolving loci. Notably, the human *DMD* gene and its mouse and rat orthologs are conserved across all three species, consistent with the fundamental role of dystrophin in muscle cell membrane integrity (Supplementary Table S3).

### 3.3. Pathway and Gene Ontology Analysis

**Figure 5:**
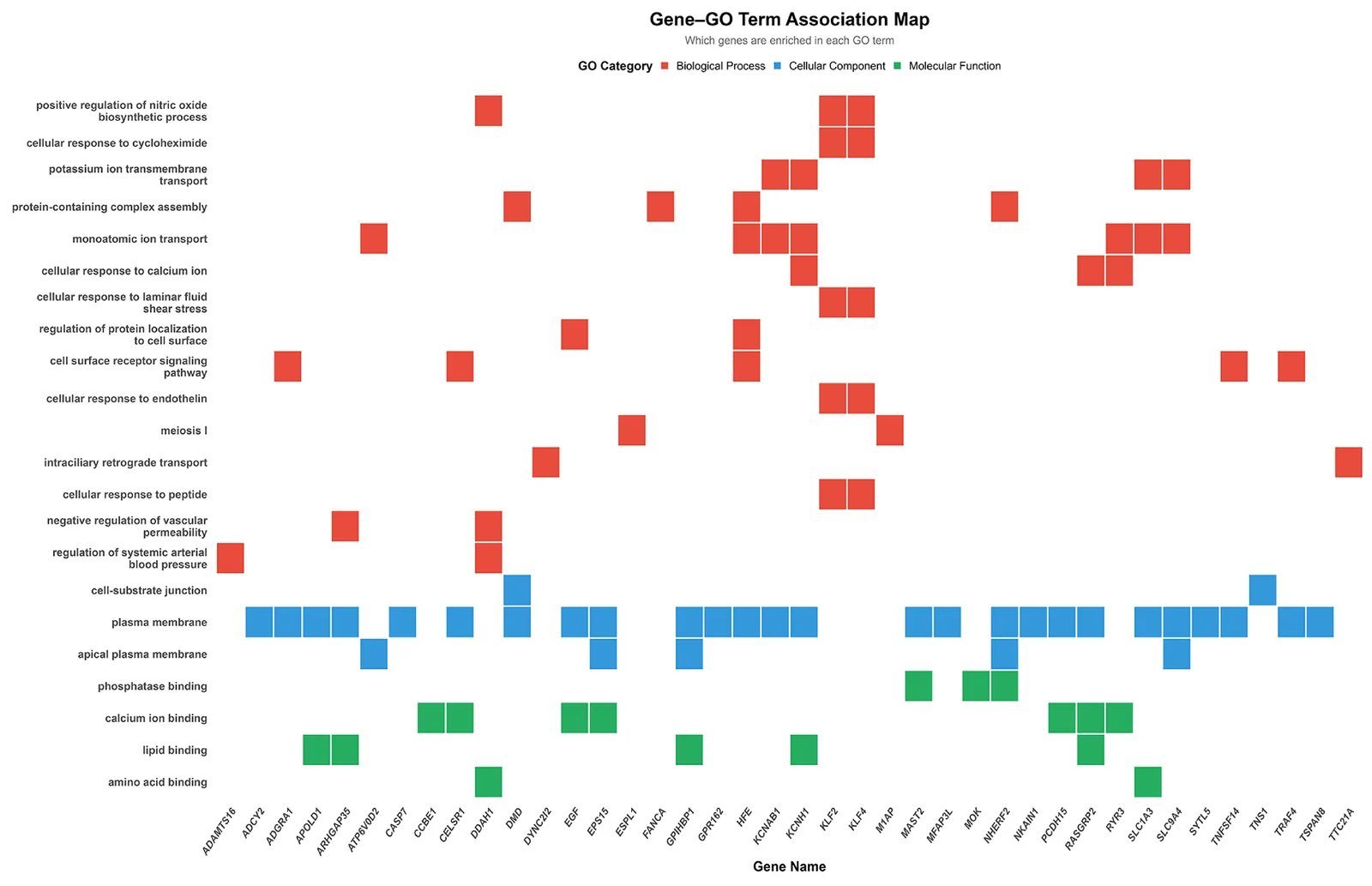
Association map linking the conserved DMD-associated genes (DAGs) to GO terms enriched in the cross-species shared DEG set. GO enrichment was performed on the shared DEG set; the 57 DAGs were subsequently mapped onto these outputs. Rows indicate GO terms containing at least one DAG, columns indicate DAGs, and colors denote GO categories: Biological Process, Cellular Component, and Molecular Function.

**Figure 6:**
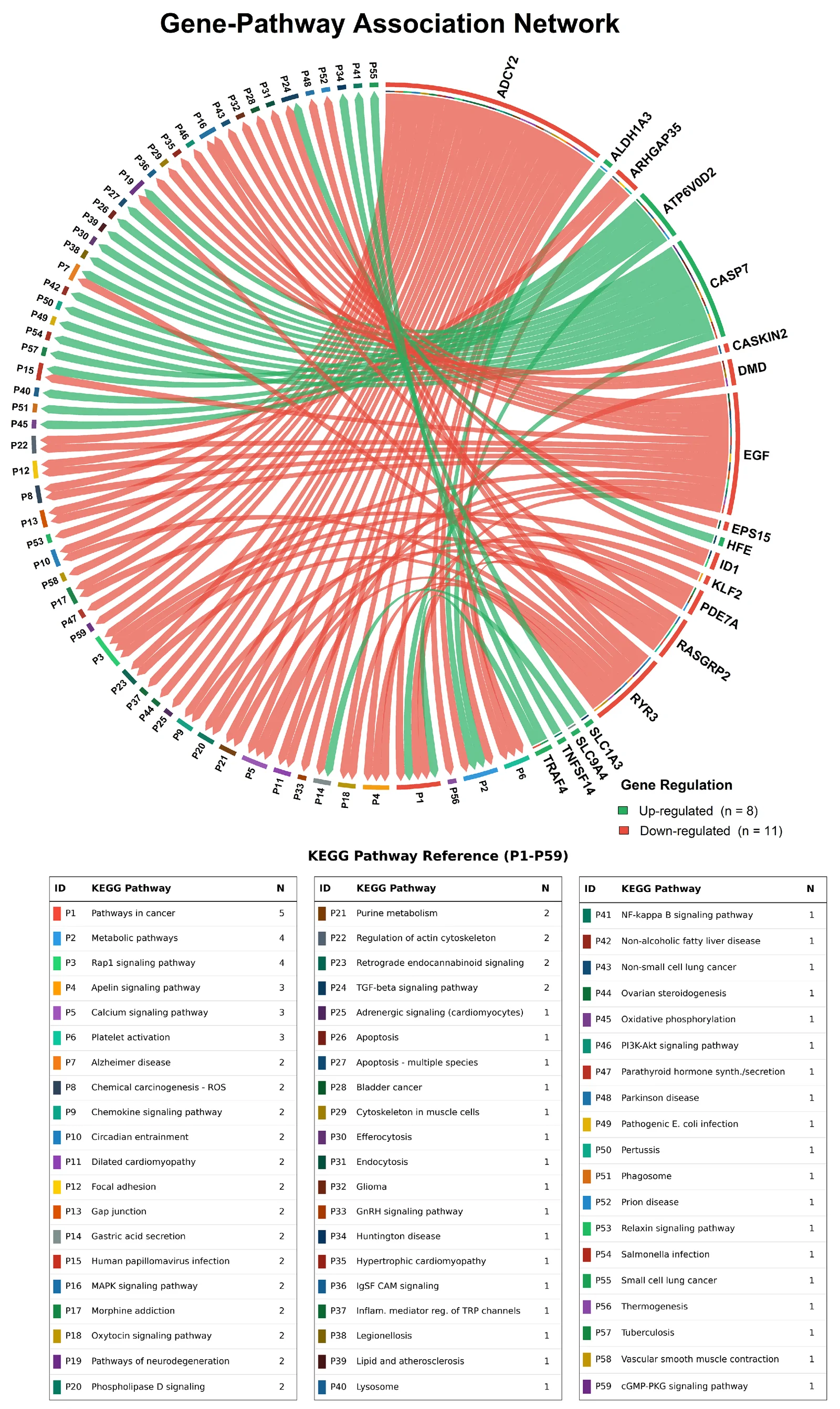
Association network linking the conserved DMD-associated genes (DAGs) to KEGG pathways enriched in the cross-species shared DEG set. KEGG enrichment was performed on the shared DEG set; the circular network shows DEG-derived pathways that contain at least one DAG and the corresponding DAGs mapped to each pathway. Green indicates upregulated genes and red indicates downregulated genes. Pathway identifiers correspond to the KEGG pathway reference table shown below the network, with N denoting the number of DAGs mapped to each pathway.

DEG-based functional enrichment analysis identified nominally enriched GO terms and KEGG pathways associated with the cross-species shared DEG set. To address pathway-level robustness, DAVID Benjamini–Hochberg FDR-adjusted enrichment statistics were added for GO and KEGG results, and the final ortholog-filtered DAGs were mapped to these enrichment outputs. Of the 60 KEGG pathways containing at least one DAG, 52 were nominally significant (*p* < 0.05) and 34 remained significant after FDR correction, including focal adhesion (FDR = 1.4 × 10^−3^), apoptosis (FDR = 0.029), and calcium signaling (FDR = 0.046). Rap1 signaling was nominally enriched (*p* < 0.05) but did not remain significant after Benjamini–Hochberg correction (FDR = 0.100) and is therefore described throughout as nominally enriched only. Of the 76 GO terms containing at least one DAG, all satisfied both nominal *p* < 0.05 and FDR < 0.05. These supplementary GO/KEGG summaries distinguish nominally enriched terms and pathways from those supported after multiple-testing correction and identify which DAGs contributed to the main functional categories (Figure 5 and Figure 6; Supplementary Tables S4 and S5). These results support the biological coherence of the pathway-level interpretation while preserving the exploratory status of the 57-gene DAG signature. Notably, classical downstream pathological categories such as fibrosis, immune response, and extracellular matrix deposition were not among the dominant DEG-derived categories to which the conserved DAGs mapped.

### 3.4. circRNAs Derived from the 57 Conserved DMD-Associated Genes

**Figure 7:**
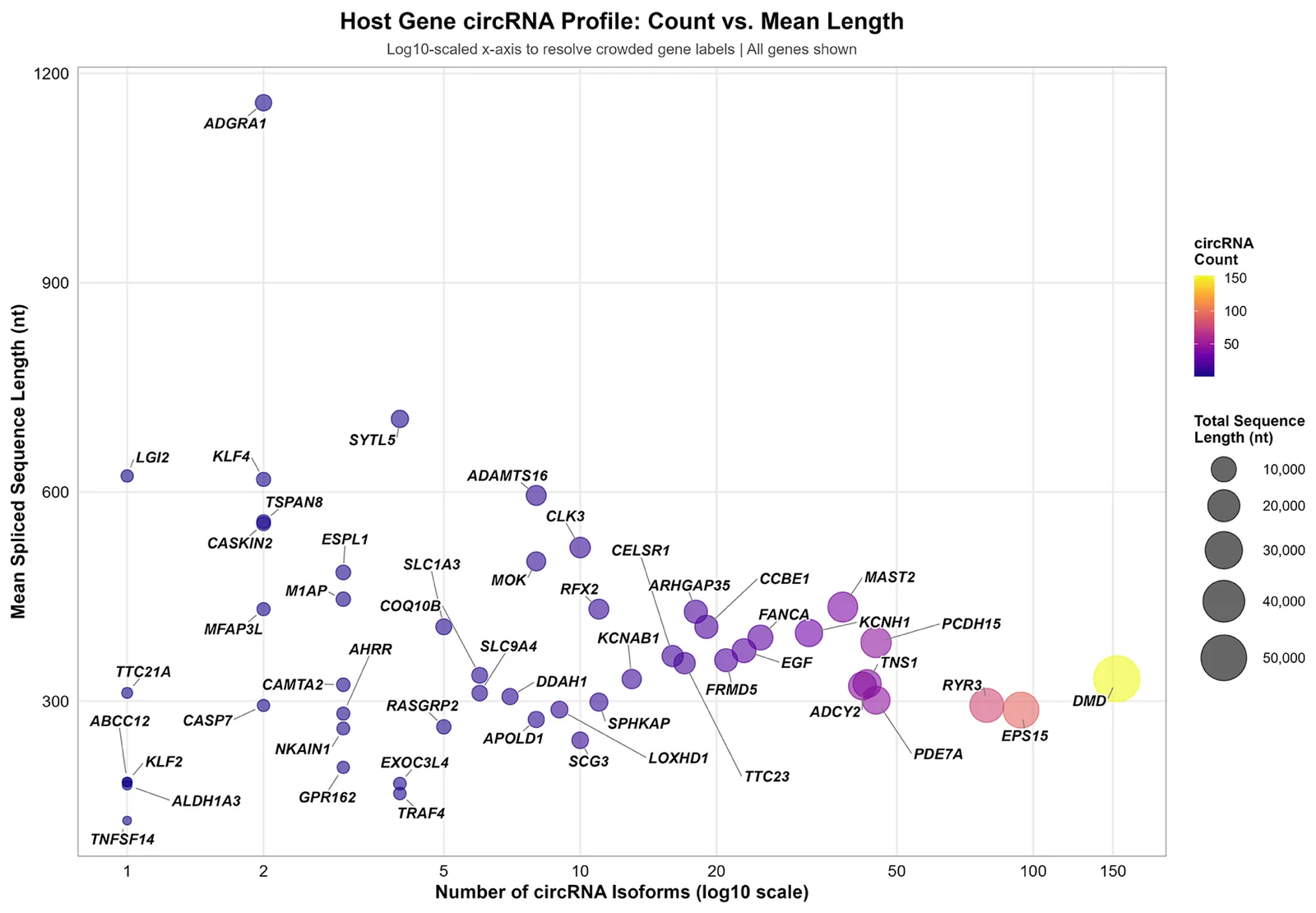
Number of annotated circRNA isoforms derived from concordantly dysregulated genes across species. Genes with large, multi-exonic genomic structures produced the highest numbers of circRNA isoforms.

circAtlas provides unified circRNA annotations for human datasets and uses hg19 as the human genome assembly. Analysis of circBase annotations showed that a substantial proportion of the 57 conserved DAGs generated annotated circRNAs. Genes producing the largest numbers of circRNA isoforms were predominantly those with large multi-exonic genomic structures. Among these loci, DMD was one of the most prominent sources of circRNA isoforms, together with *RYR3*, *PCDH15*, *MAST2*, *CELSR1*, and *ADAMTS16* (Figure 7).

### 3.5. Predicted Direct circRNA–miRNA Interactions

To identify candidate miRNA-binding circRNAs, circRNA sequences were retrieved from circAtlas and screened against the 7-mer seed regions of all 2656 human mature miRNAs. This analysis identified 3211 circRNA–miRNA pairs with at least two seed-matching sites, involving 456 unique circRNAs and 1281 unique miRNAs (Supplementary Table S6). Most retained pairs contained two binding sites, whereas a smaller subset contained three or more binding sites, consistent with stronger putative sponge activity. Overall, longer circRNAs tended to interact with larger numbers of miRNAs.

### 3.6. miRNA Targets and Integrated ceRNA Network

miRNA–target interaction records for the 57 conserved DAGs were retrieved using multiMiR (Supplementary Table S7). All circRNA–miRNA and miRNA–mRNA interactions reported in this network represent computationally predicted or database-curated associations; circRNA and miRNA expressions were not measured in the analyzed datasets, and expression correlation analysis was not performed. miRNA regulators were identified for most genes in the set, and several genes showed particularly dense miRNA regulation, including *KLF4*, *EGF*, *ID1*, *DMD*, and *CASP7*. For example, hsa-miR-125b-5p was identified as a regulator of *CASP7*, whereas hsa-miR-10b-5p and hsa-miR-137-3p were identified as regulators of *KLF4*. The retrieved multiMiR records also included several muscle-enriched miRNAs, including hsa-miR-1-3p, hsa-miR-206, hsa-miR-133a-3p, and hsa-miR-486-5p, connected with conserved DMD-associated genes such as *DMD*, *KLF4*, *TNS1*, *MAST2*, *ARHGAP35*, *EPS15*, and *PDE7A*. In addition, several miRNA families targeted multiple DMD-associated genes, indicating convergent post-transcriptional regulation within the network. Integration of predicted circRNA–miRNA interactions with miRNA–gene pairs yielded a tripartite circRNA–miRNA–mRNA ceRNA network. In ceRNA models, circRNAs are commonly interpreted as potential miRNA sponges. The most connected circRNAs were largely derived from large conserved genes and were linked to multiple DMD-associated genes through shared miRNA intermediates. The highest-ranked circRNAs showed extensive overlap in targeted miRNAs across multiple DMD-associated genes, indicating the presence of dense regulatory subnetworks. Notably, circRNAs derived from *DMD* itself were connected to miRNAs targeting other DMD-associated genes, including *TIMP4*, *KLF4*, and *ID1*, suggesting a possible intra-network feedback architecture.

### 3.7. Candidate Regulatory circRNAs Derived from Conserved Host Genes

Most circRNAs identified in the inferred ceRNA network originated from host genes with confirmed orthologs in humans, mice, and rats, indicating that the predicted circRNA–miRNA–mRNA network was largely organized around evolutionarily conserved DMD-associated host genes. A subset of these candidate regulatory circRNAs was generated directly from the 57 conserved DAGs, creating a self-referential network in which circRNAs derived from DMD-related genes potentially modulate miRNAs that target other members of the same gene set. This pattern is consistent with the ceRNA model of coordinated regulation among functionally related transcripts.

### 3.8. Network Topology and Major Regulatory Hubs

**Figure 8:**
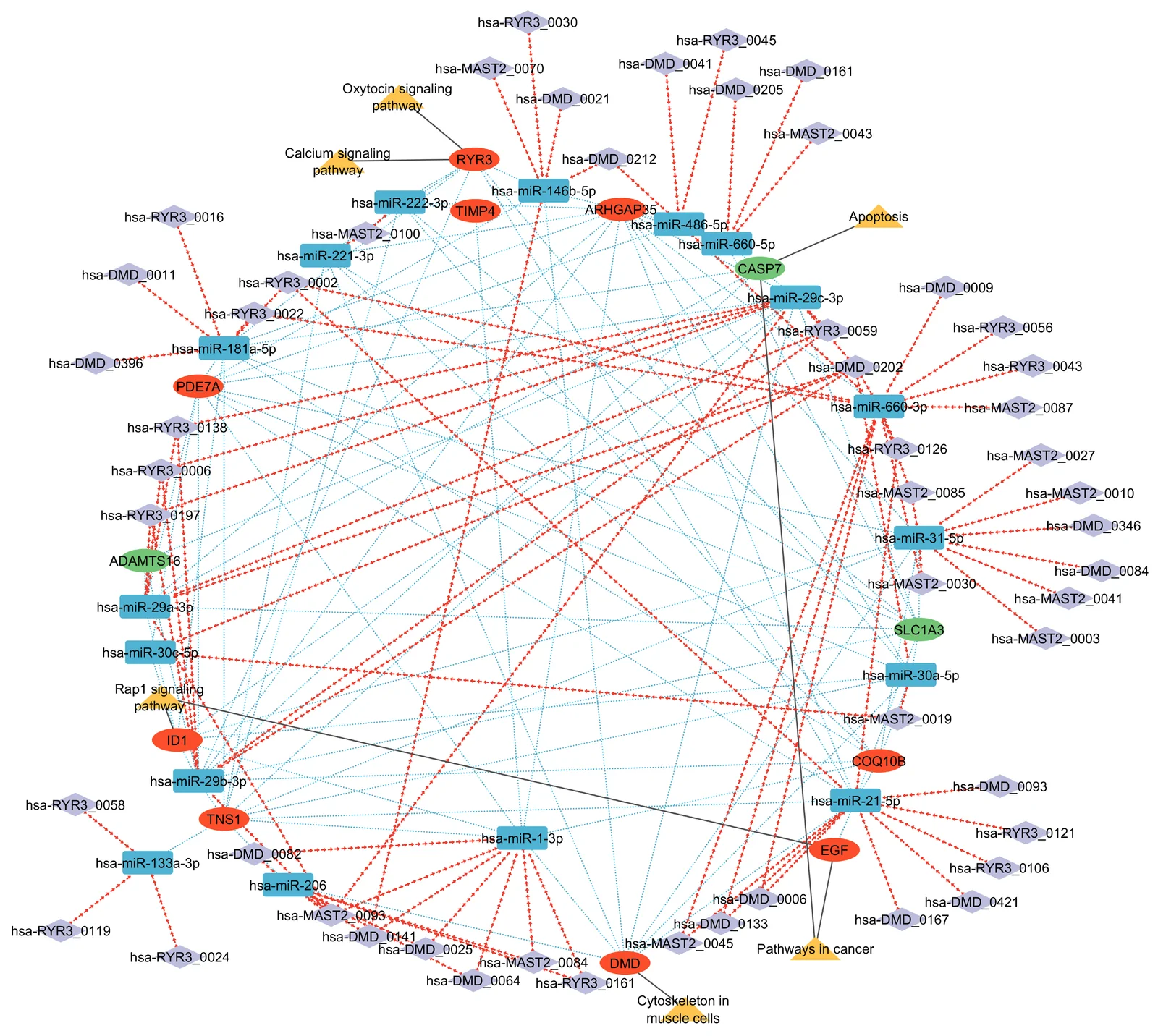
The integrated candidate circRNA–miRNA–mRNA ceRNA network of DMD-associated genes shows a hub-dominated topology. Key miRNAs regulate multiple targets, while circRNA hubs are modeled as putative sponges within dense regulatory subnetworks. Node colors/shapes: red circles (downregulated genes), green circles (upregulated genes), blue rectangles (miRNAs), purple diamonds (circRNAs), and yellow triangles (pathways). Red dashed lines denote predicted miRNA–circRNA sponging interactions, blue dotted lines denote miRNA–target gene interactions, and black solid lines connect genes to their enriched pathways.

Network visualization in Cytoscape showed a strongly hub-dominated topology, with a limited number of circRNAs and miRNAs accounting for a large proportion of network connectivity. Highly connected miRNAs targeted multiple DMD-associated genes and may represent candidate regulatory bottlenecks. Conversely, highly connected circRNAs interacted with broad sets of miRNAs, suggesting they are candidate regulatory hubs for future validation (Figure 8).

## 4. Discussion

### 4.1. A Conserved Molecular Core of Dystrophin-Deficient Muscle

**Figure 9:**
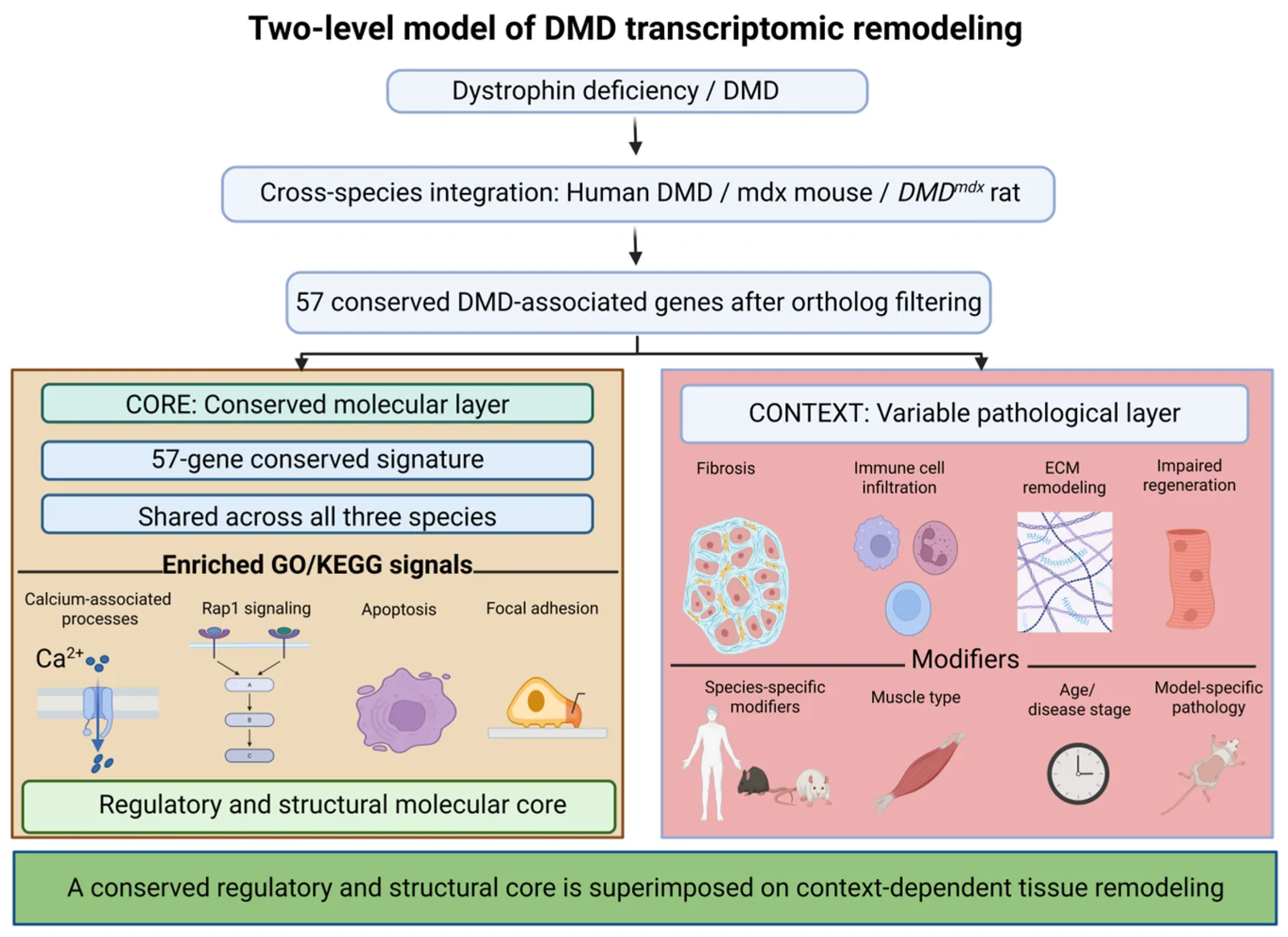
CORE vs CONTEXT model of DMD-associated transcriptomic remodeling. Cross-species integration of human DMD, mdx mouse, and *DMD^mdx^* rat skeletal muscle datasets identified a conserved 57-gene molecular core. Functional enrichment was performed on the cross-species shared DEG set, and mapping of the DAGs onto these outputs highlighted calcium-associated processes, apoptosis, and focal adhesion, which remained significant after FDR correction, together with nominally enriched Rap1 signaling. Classical pathological features such as fibrosis, immune-cell infiltration, ECM remodeling, and regenerative failure are proposed as more context-dependent responses shaped by species, muscle type, age, disease stage, and model-specific pathology.

This study identifies a conserved 57-gene transcriptional signature shared across human DMD skeletal muscle and dystrophin-deficient mouse and rat muscle, supporting a recurrent cross-species molecular response to dystrophin deficiency. Importantly, this signature does not map to a single pathogenic pathway; rather, it reflects integrated tissue-level remodeling involving sarcolemmal instability, cytoskeletal and adhesion changes, calcium and cAMP/PKA-associated signaling, immune-lysosomal activity, ECM turnover, impaired regeneration, endothelial dysfunction, and secondary metabolic stress [10,23,24,25]. The enrichment results provide an important clue for interpreting the conserved signature. Because enrichment was computed on the cross-species shared DEG set and the 57 conserved DAGs were subsequently mapped onto these outputs, the pattern described here reflects the DEG-derived categories in which conserved genes are represented rather than enrichment of the 57-gene set itself. Although fibrosis, immune infiltration, and ECM accumulation are major hallmarks of DMD pathology, the conserved DAGs mapped only sparsely onto these categories. Instead, they mapped preferentially onto calcium-related processes, apoptosis, and focal adhesion, which remained significant after FDR correction, and onto Rap1 signaling, which was nominally enriched only. This supports a two-level interpretation of DMD transcriptomic remodeling, in which a conserved regulatory and structural core is superimposed on context-dependent responses shaped by species, muscle type, age, disease stage, and model-specific pathology (Figure 9).

This interpretation also helps explain why several classical DMD pathology markers, such as *COL1A1*, *MMP2*, *IL6*, and *SPP1*, were not retained within the conserved 57-gene DAG set. These genes are strongly linked to fibrosis, extracellular matrix remodeling, inflammation, and macrophage-associated tissue responses, but such processes are highly sensitive to disease stage, muscle type, species background, and the severity of secondary tissue remodeling. Their absence from the conserved core, therefore, does not imply biological irrelevance. Rather, their absence from the conserved core is consistent with greater context dependence, whereas the 57-gene signature captures recurrent regulatory, structural, calcium-associated, apoptotic, and adhesion-related components shared across the analyzed datasets.

The observed downregulation of DMD should be interpreted cautiously, as reduced dystrophin expression is expected in dystrophin-deficient tissues rather than representing a novel downstream discovery. Nevertheless, its presence anchors the conserved transcriptomic profile to the primary pathogenic context of DMD, in which dystrophin loss is known to destabilize the sarcolemma and is associated with repeated cycles of myofiber injury, inflammation, and incomplete regeneration [10,23,24]. Because the analysis was based on bulk RNA-seq, the identified signature should not be considered exclusively myofiber-derived. Based on previous single-cell and single-nucleus studies, dystrophic muscle is expected to contain multiple remodeling cell populations, including fibro-adipogenic progenitors, fibroblasts, endothelial cells, pericytes, macrophages, and other immune populations, which undergo major compositional and state changes in DMD [26,27,28]. Accordingly, the 57-gene DAG signature reflects tissue-level transcriptional remodeling in dystrophic skeletal muscle. Functional and cellular interpretations of this signature should therefore be viewed as literature-supported hypotheses derived from transcriptomic patterns rather than as direct functional validation.

### 4.2. Structural and Calcium-Related Anchors of DMD Biology

A major component of this conserved program involves remodeling of the sarcolemma–cytoskeleton–ECM interface. Reduced expressions of *TNS1*, *ARHGAP35*, *NHERF2*, *MAST2*, *EPS15*, and *TSPAN8* suggest altered focal adhesion integrity, cytoskeletal scaffolding, Rho/Rap-associated signaling, and membrane trafficking. *TNS1* is noteworthy because tensins couple integrin adhesion complexes to the actin cytoskeleton and contribute to cell adhesion and mechanotransduction [29]. Thus, reduced expressions of these genes may indicate impaired cell–matrix coupling and altered mechanosensing in dystrophic muscle. Conversely, increased expressions of *ADGRA1*, *PCDH15* [30], *CELSR1* [31], *FRMD5* [32], and *GPR162* point to alternative adhesion, polarity, and tissue-organization programs. Based on the known roles of these genes in prior literature, this pattern may reflect compensatory remodeling, altered intercellular communication, or shifts in non-myogenic cell populations.

The signature also implicates calcium-associated, cAMP/PKA, and membrane-excitability pathways. Downregulation of *RYR3*, *ADCY2*, *PDE7A*, *KCNAB1*, and *NKAIN1*, together with upregulation of *SPHKAP*, *KCNH1*, *SLC9A4*, and *SLC1A3*, suggests altered signaling and ion-handling programs rather than direct evidence of measured calcium flux abnormalities [33,34,35]. Notably, the simultaneous decrease in *ADCY2* and *PDE7A* argues against a simplistic interpretation of cAMP signaling as either activated or suppressed; instead, it suggests reorganization of compartmentalized cAMP turnover, supported by increased SPHKAP, an A-kinase anchoring protein involved in localized PKA signaling [36]. Additional genes in this axis, including *CAMTA2*, *CASKIN2*, *CLK3*, *LGI2*, and *LOXHD1*, are not canonical DMD markers, but their conserved dysregulation suggests broader involvement in transcriptional, scaffolding, and possibly sensory or neuronal-like signaling.

### 4.3. Context-Dependent Remodeling of the Dystrophic Muscle Microenvironment

Another feature of the shared signature is immune–lysosomal and tissue-clearance remodeling. Chronic inflammation is a defining component of DMD pathology, and inflammatory signaling in mdx muscle contributes to disease progression rather than simply reflecting tissue damage [37,38]. Within this axis, upregulation of *ATP6V0D2* suggests enhanced macrophage/lysosomal activity associated with phagocytic clearance of damaged fibers and altered autophagy–lysosomal function in dystrophic muscle [39,40]. Concurrent upregulation of *TNFSF14*, *TRAF4*, *CASP7*, *HFE*, and *AHRR* further supports inflammatory, stress-related, and immunometabolic remodeling. CASP7 may indicate engagement of cell-death-related pathways, although in dystrophic muscle, this should be interpreted within a broader tissue-damage context involving necrosis, inflammatory clearance, and degradation of damaged structures [41].

The conserved signature additionally highlights dynamic ECM turnover, fibrosis-associated remodeling, and dysregulated regeneration as major components of dystrophic muscle biology. Rather than representing a static collagen-dominated fibrotic profile, the transcriptomic pattern suggests active and imbalanced matrix remodeling, marked by upregulation of *ADAMTS16* and downregulation of *TIMP4*, *MFAP3L*, and *TNS1*. Because *ADAMTS16* has been linked to ECM remodeling and TGF-β-associated fibrosis, whereas TIMPs regulate metalloproteinase activity, their opposing regulation is consistent with heightened matrix turnover [42,43]. Additional genes, including *CCBE1*, *FRMD5*, *CELSR1*, *ADGRA1*, and *RASGRP2*, suggest that cell–matrix signaling, stromal organization, and vascular or lymphatic remodeling are altered in parallel.

This ECM remodeling state appears to be coupled to a dysregulated regenerative niche. DMD muscle undergoes continuous cycles of attempted repair, but regenerative capacity progressively fails as inflammation and fibrosis intensify [23,44,45]. Downregulation of ID1 and EGF, together with upregulation of *ESPL1*, *FANCA*, *ALDH1A3*, *OSR2*, and *MOK*, suggests altered regulation of progenitor cells, proliferative activity, differentiation control, and stress-response pathways. Accordingly, the regeneration-related interpretation should be regarded as a literature-supported hypothesis derived from the conserved expression pattern rather than as direct evidence from cellular, histological, or functional regenerative assays. Reduced *ID1* may indicate altered BMP/ID-mediated regulation of progenitor proliferation and myogenic differentiation, whereas increased *ESPL1* and *FANCA* may reflect proliferative and stress-response activity in remodeling cells [46]. Together, these findings support the concept of a dystrophic repair niche in which regeneration, fibrosis, stromal activation, and tissue reorganization coexist but fail to restore durable muscle integrity.

The conserved program also underscores an endothelial, microvascular, and metabolic component of DMD pathology. Although DMD originates from dystrophin deficiency in myofibers, disease progression is influenced by impaired nitric oxide signaling, functional ischemia, and microvascular abnormalities [47,48], and single-nucleus studies support endothelial transcriptional remodeling in dystrophic muscle [25]. Downregulation of *APOLD1*, *KLF2*, *KLF4*, *GPIHBP1*, and *RASGRP2*, together with upregulation of *DDAH1* and *CCBE1*, suggests altered endothelial homeostasis, vascular remodeling, and capillary metabolic function. Together with *COQ10B*, *HFE*, and *ALDH1A3*, these changes indicate that vascular dysfunction intersects with oxidative and metabolic stress in dystrophic muscle. These changes point to vascular and metabolic remodeling as part of the conserved tissue-level signature, while dedicated histological or physiological studies would be required to assess endothelial function directly.

Together, these findings define DMD as a multicellular tissue-remodeling state rather than a purely structural myopathy. The integration of sarcolemmal instability, immune–lysosomal activity, ECM turnover, regenerative failure, endothelial dysfunction, and metabolic stress provides a coherent framework for interpreting conserved and context-dependent disease progression across species.

### 4.4. Post-Transcriptional and Pathway-Level Integration of the Conserved DMD Network

The miRNA findings add an important post-transcriptional regulatory dimension to the DMD network. In our miRNA–target interaction analysis, miRNA regulation was particularly evident for genes such as *CASP7* and *KLF4*, including interactions involving hsa-miR-125b-5p, hsa-miR-10b-5p, and hsa-miR-137-3p, supporting convergent post-transcriptional control within the network. Muscle-enriched miRNAs, including miR-1-3p, miR-206, miR-133a-3p, and miR-486-5p, further support the involvement of myogenic and regenerative regulatory programs in the conserved DMD network. These myomiRs have been repeatedly implicated in muscle differentiation, regeneration, and dystrophic pathology [49,50]. Additional miRNAs, including miR-30c-5p, miR-181a-5p, miR-146b-5p, and miR-660-5p, may reflect inflammatory adaptation, cell-state transitions, and stress signaling superimposed on this myogenic regulatory layer.

CircRNAs are stable regulatory RNAs that can modulate gene expression through interactions with miRNAs and RNA-binding proteins, making them relevant to diseases in which persistent stress and tissue remodeling reshape transcriptional networks over time [51,52]. The identification of DMD- and RYR3-derived circRNAs is especially noteworthy because their parental genes are central to the structural and calcium-related interpretation of DMD biology. Likewise, MAST2-derived circRNAs may reflect a broader cytoskeletal-signaling context. Together, these findings suggest that circRNA-associated regulation may be linked to structural, calcium-related, and cytoskeletal-signaling components of the dystrophic muscle transcriptome.

The profile of DEG-derived pathways onto which the conserved DAGs mapped further reinforces this integrated interpretation. Oxytocin signaling may initially appear unexpected, but it intersects with calcium-dependent and MAPK-related processes relevant to muscle adaptation and regeneration [53]. Rap1 signaling, although supported at the nominal level only (FDR = 0.100), is likewise biologically plausible because it links adhesion and cytoskeletal dynamics to cell-survival and remodeling responses [54]. It is therefore discussed as a candidate rather than as an FDR-supported pathway. Together, these pathways support a model in which structural instability, calcium-associated signaling, apoptosis, RNA-mediated regulation, and maladaptive repair interact within the dystrophic muscle transcriptome. From a translational perspective, the most relevant cross-species candidates are those bridging these domains, including DMD, RYR3, CASP7, myomiRs, and DMD- and RYR3-derived circRNAs.

### 4.5. Cross-Species Transcriptomic Discordance and Model-Specific Pathology

The integrative strategy used in this study—the overlap of independently derived, ortholog-mapped DEGs rather than joint cross-species statistical modeling—was adopted specifically to accommodate the substantial technical and biological heterogeneity across the three datasets, including differences in sequencing platform, reference genome annotation, library preparation, muscle type, age, and disease stage. Because each dataset was analyzed independently within its own experimental design, cross-study batch effects are not directly propagated into the conserved gene set; however, they remain a relevant source of the discordance observed for genes that were not recovered across all three species. Study-specific biases, including differences in sequencing depth, sample size, and control-group composition, may similarly influence which genes reach statistical significance within each species, contributing to apparent cross-species divergence that reflects technical rather than purely biological variation.

The consistent downregulation of DMD across human, mouse, and rat datasets, together with species-dependent expression of several secondary-response genes, is biologically plausible. In cross-species DMD analyses, limited overlap and even opposite-direction differential expressions are expected for genes involved in fibrosis, inflammation, regeneration, and ECM remodeling, because these processes are strongly influenced by species, muscle type, and disease stage.

This divergence reflects intrinsic species-specific modifiers and compensatory mechanisms. The classical mdx mouse exhibits a relatively mild phenotype, with efficient regeneration, limited fibrosis, and a near-normal lifespan, partly due to compensatory upregulation of utrophin [55,56]. In contrast, the *DMD^mdx^* rat displays a more severe and progressive dystrophic phenotype, with persistent inflammation, impaired regeneration, FAP expansion, and extensive fibrosis, more closely resembling key aspects of human DMD pathology [10,57]. Less effective utrophin compensation in rats may further contribute to this greater disease severity [58,59,60]. Thus, interspecies transcriptomic differences likely reflect both the primary *DMD* defect and broader genetic background effects, compensatory mechanisms, and species-specific regulatory networks [61].

### 4.6. Muscle Type and Temporal Heterogeneity in Cross-Species Comparisons

Muscle type is an important source of heterogeneity in cross-species transcriptomic comparisons. The present study integrates human tibialis anterior, mouse gastrocnemius, and rat biceps femoris datasets, representing muscles with distinct fiber-type compositions and susceptibilities to dystrophic remodeling [62,63]. Because fast-twitch-dominant muscles are generally more vulnerable to DMD-associated degeneration, and slow- versus fast-twitch fiber proportions vary between species and muscle groups, local transcriptomic responses are expected to differ [64,65]. Therefore, divergent expressions of genes such as *SPP1*, *UTRN*, *COL1A1*, and *MMP2* may reflect muscle-type-specific remodeling rather than fundamental biological contradiction [24,66,67].

Disease stage is another major source of transcriptomic heterogeneity. In mdx mice, necrosis and regeneration peak early and are followed by a plateau phase with limited fibrosis until advanced age, whereas *DMD^mdx^* rats show early inflammation and fibrosis that progress more rapidly and more closely parallel key aspects of human disease [68]. Human DMD develops over the years, with chronic fibrosis and progressive regenerative failure [69]. Because the analyzed datasets were collected at different points along these species-specific disease timelines, the dominant pathological process at sampling may differ, producing stage-dependent differences or even reversals in secondary gene-expression patterns.

Collectively, muscle type, disease stage, and study-specific technical factors represent important sources of heterogeneity that are not resolved by ortholog overlap alone; rather, the conserved 57-gene DAG signature should be interpreted as the subset of the transcriptomic response that remains detectable despite this heterogeneity, while genes that fail to reach conservation across all three datasets may reflect either genuine species-specific biology or the influence of batch- and study-level variability.

### 4.7. Novelty, Limitations, and Interpretative Framework

The human, mouse, and rat datasets differ in muscle type, age, disease stage, and platform, making the conservation-based strategy central to the interpretation of the present analysis. A gene concordantly dysregulated across three heterogeneous, independent datasets is unlikely to reflect a dataset-specific artifact; rather, its recurrence supports a conserved molecular response associated with dystrophin deficiency. The 57-gene signature therefore represents the component of the transcriptomic response retained after cross-species filtering, whereas non-retained genes are more likely to reflect context-dependent biology and/or batch- and study-level variability. This study differs from previous DMD transcriptomic analyses by integrating human DMD skeletal muscle with two dystrophin-deficient animal models, the mdx mouse and the *DMD^mdx^* rat. By applying ortholog filtering, we identified a conserved cross-species transcriptional core and interpreted it in relation to broader species- and model-dependent responses. This approach distinguishes recurrent molecular features of dystrophin deficiency from secondary pathological processes shaped by species, muscle type, age, disease stage, and model severity.

A further novel aspect is the integration of the conserved 57-gene DAG signature with circRNA- and miRNA-based regulatory analyses. This enabled the construction of a candidate ceRNA framework centered on conserved DMD-associated host genes and highlighted post-transcriptional regulatory layers involving circRNAs, miRNAs, and DMD-associated mRNAs. Although hypothesis-generating, this framework provides a structured basis for prioritizing conserved regulatory candidates for future validation.

Several limitations should be considered. The human dataset includes an age mismatch between DMD patients and healthy controls, which may contribute to part of the observed transcriptional differences; therefore, the conserved signature should be interpreted as DMD-associated rather than strictly DMD-specific. The FDR-based DEG sensitivity analysis showed that only 7 of the 62 directionally concordant genes remained supported after multiple-testing correction. Because this subset comprises only seven genes, a formal enrichment analysis restricted to it would have limited statistical power and interpretability; such an analysis was therefore not performed and was not used to draw pathway-level conclusions. Instead, the seven genes are described in terms of their mapping onto the DEG-derived enrichment outputs. At the pathway level, this reduced FDR-supported gene subset provided limited coverage of the broader GO/KEGG profile: four of the seven genes (*DMD*, *CASKIN2*, *KLF2*, and *ID1*) were annotated to at least one category that remained significant after FDR correction in the shared DEG set, whereas *CCBE1*, *RFX2*, and *KLF4* were annotated only to categories that were nominally enriched. In the case of *ID1*, this FDR-supported annotation derives from the GO categories listed in Supplementary Table S4; the Rap1 signaling pathway, to which *ID1* is also annotated, was nominally enriched only (FDR = 0.100) and is not counted as FDR-supported. Thus, the DEG-level FDR analysis supports a smaller statistically stringent subset within the conserved signature, whereas the broader 57-gene DAG signature and its associated regulatory network remain hypothesis-generating. Future studies with larger sample sizes will be needed to validate this signature under more stringent statistical criteria. This study relied entirely on computational integration of publicly available RNA-seq datasets, and we did not have access to independent samples or datasets for experimental or computational validation. In addition, because circRNA annotations and circRNA–miRNA interactions were inferred computationally, the analysis supports conservation of circRNA host genes rather than direct conservation of functional circRNA-mediated regulation across species.

## 5. Conclusions

This study supports a two-level model of DMD-associated transcriptomic remodeling. Reduced DMD expression served as a molecular anchor across human, mouse, and rat datasets, whereas pathological features related to fibrosis, inflammation, regeneration, and extracellular matrix remodeling were interpreted as more context-dependent responses shaped by species, muscle type, age, disease stage, and model-specific pathology (Figure 9).

We identified 1367 shared differentially expressed genes, of which 62 showed concordant directionality across all three species and 57 were retained after ortholog filtering. Functional enrichment was performed on the cross-species shared DEG set, and mapping of the conserved DAGs onto these outputs highlighted calcium-related processes, apoptosis, and focal adhesion, which remained significant after FDR correction, together with nominally enriched Rap1 signaling. This supports a conserved regulatory and structural core rather than a complete representation of all classical DMD pathological features. Integration with circRNA- and miRNA-based regulatory analyses identified a candidate, computationally inferred hub-dominated ceRNA network and prioritized candidate regulatory relationships.

Overall, these findings distinguish recurrent cross-species molecular components from more variable secondary responses, improving the interpretation of DMD animal models and prioritizing *RYR3*, *CASP7*, *KLF4*, *ID1*, and *TIMP4*, along with DMD- and RYR3-derived circRNAs, as candidates for future mechanistic and translational studies. Within this signature, seven genes (*CCBE1*, *RFX2*, *CASKIN2*, *DMD*, *ID1*, *KLF2*, and *KLF4*) were retained under FDR-adjusted differential expression criteria.

## Figures and Tables

**Table 1:** Summary of RNA-seq datasets used in this study, including species, sample types, experimental groups, age ranges, and corresponding public database accession numbers. “Duration” indicates the length of the experimental treatment period (i.e., the time animals were maintained under the specified disease-model condition prior to tissue collection); this parameter was not applicable to the human dataset (“–”), as human samples were obtained cross-sectionally.

Species	Sample Types	Experimental Groups	Age Range	Duration	Dataset References
Human	Tibialis anterior muscle	Healthy, DMD, DMD 6311, Controls	Healthy (19–24 years) DMD (2–7 years)	–	PRJNA976807
Mouse	Gastrocnemius muscle	mdx, Healthy, C57BL/10ScSn-Dmd*^mdx^*	7 weeks	15 weeks (in vivo)	PRJNA1009848
Rat	Biceps femoris muscle	*DMD^mdx^*, WT control	13–14 weeks	6 weeks	PRJNA1191685

## Data Availability

All RNA-seq datasets analyzed in this study are publicly available in the NCBI Sequence Read Archive (SRA). The human and mouse datasets were retrieved under BioProject accession numbers PRJNA976807 and PRJNA1009848, respectively. The rat *DMD^mdx^* RNA-seq dataset, previously generated by the authors within the same research project and reanalyzed here as part of the cross-species integration, is available under BioProject accession number PRJNA1191685. No new sequencing data were generated for the present integrative analysis. Grammarly AI software, version 1.2.288, was used to improve the quality of the English language.

## Supplementary Materials

The following supporting information can be downloaded at https://www.mdpi.com/article/10.3390/genes17091017/s1; Supplementary Table S1: Complete differential expression analysis results (DESeq2 v1.46.0) for each species. (A) Human tibialis anterior, DMD (*n* = 8) vs. healthy control (*n* = 5), BioProject PRJNA976807; (B) mouse gastrocnemius, C57BL/10ScSn-Dmdmdx (*n* = 5) vs. BL10 control (*n* = 5), BioProject PRJNA1009848; (C) rat biceps femoris, *DMD^mdx^* (*n* = 4) vs. wild-type control (*n* = 4), BioProject PRJNA1191685. Log2 fold change is expressed as DMD vs. control. Genes meeting the primary exploratory screening thresholds (nominal *p* < 0.05 and |log2FC| > 0.3) are indicated and correspond to the colored points in Figure 2. Supplementary Table S2: Sensitivity analysis of differentially expressed genes using Benjamini–Hochberg FDR-adjusted *p*-values (padj < 0.05). Includes the FDR-supported DEG list per species, the pairwise cross-species overlaps under this stricter criterion (human–mouse: 146 upregulated, 58 downregulated; mouse–rat: 5 upregulated, 12 downregulated; rat–human: 6 upregulated, 22 downregulated), and the seven directionally concordant three-species genes retained after ortholog filtering (*CCBE1* and *RFX2*, upregulated; *CASKIN2*, *DMD*, *ID1*, *KLF2*, and *KLF4*, downregulated). Supplementary Table S3: Cross-species ortholog mapping of the 62 concordantly dysregulated genes using NCBI Homolo-Gene. Reports the *H. sapiens*, *M. musculus*, and *R. norvegicus* identifiers for the 57 genes (87.1%) with confirmed one-to-one orthologs in both non-human species, and flags the five genes (*EXOC3L4*, *ABCC12*, *M1AP*, *DCAF12L2*, and *INAVA*) lacking high-confidence orthologs in one or both species. Supplementary Table S4: Gene Ontology enrichment summary (DAVID) for the cross-species shared DEG set, mapped to the final ortholog-filtered DMD-associated genes (DAGs). Reports term identifier, category (Biological Process, Cellular Component, Molecular Function), gene count, nominal *p*-value, Benjamini–Hochberg FDR, and the contributing DAGs. All 76 GO terms containing at least one DAG satisfied both nominal *p* < 0.05 and FDR < 0.05. Related to Figure 5. Supplementary Table S5: KEGG pathway enrichment summary (DAVID) for the cross-species shared DEG set, mapped to the final ortholog-filtered DAGs. Reports pathway identifier and name, gene count, nominal *p*-value, Benjamini–Hochberg FDR, and the contributing DAGs. Of the 60 KEGG pathways containing at least one DAG, 52 were nominally significant (*p* < 0.05) and 34 remained significant after FDR correction, including focal adhesion (FDR = 1.4 × 10^−3^), apoptosis (FDR = 0.029), and calcium signaling (FDR = 0.046). Related to Figure 6. Supplementary Table S6: Predicted circRNA–miRNA interactions obtained by screening circAtlas circRNA sequences against the 7-mer seed regions of all 2656 human mature miRNAs. Lists the 3211 circRNA–miRNA pairs retained with at least two seed-matching sites, comprising 456 unique circRNAs and 1281 unique miRNAs, together with the number of binding sites per pair and the circRNA host gene. All interactions are computationally predicted; circRNA and miRNA expression levels were not measured. Predicted circRNA–miRNA interactions identified by 7-mer seed-region screening of circAtlas circRNA sequences. Supplementary Table S7: miRNA–target interaction records for the 57 conserved DAGs retrieved with the multiMiR R package, comprising validated and predicted miRNA–mRNA associations together with their supporting source databases. These records were integrated with the circRNA–miRNA pairs in Table S6 to construct the tripartite ceRNA network shown in Figure 8. No expression correlation analysis was performed.

## Abbreviations

The following abbreviations are used in this manuscript:

Abbreviation	Full Name/Definition
ADAMTS16	ADAM Metallopeptidase with Thrombospondin Type 1 Motif 16
ADGRA1	Adhesion G Protein-Coupled Receptor A1
AHRR	Aryl Hydrocarbon Receptor Repressor
ALDH1A3	Aldehyde Dehydrogenase 1 Family Member A3
APOLD1	Apolipoprotein L Domain Containing 1
ATP6V0D2	ATPase H+ Transporting V0 Subunit D2
Biostrings	Biological Strings (R/Bioconductor package)
CAMTA2	Calmodulin Binding Transcription Activator 2
CASKIN2	CASK Interacting Protein 2
CASP7	Caspase 7
CCBE1	Collagen and Calcium Binding EGF Domains 1
CELSR1	Cadherin EGF LAG Seven-Pass G-Type Receptor 1
ceRNA	Competing Endogenous RNA
circRNA	Circular RNA
CLK3	CDC Like Kinase 3
COMP	Cartilage Oligomeric Matrix Protein
COQ10B	Coenzyme Q10B
Cytoscape	Network Visualization Software
DDAH1	Dimethylarginine Dimethylaminohydrolase 1
DEG	Differentially Expressed Gene
DESeq2	Differential Expression Analysis Tool (R package)
DMD	Duchenne Muscular Dystrophy
dplyr	Data Manipulation Package in R
ECM	Extracellular Matrix
EGF	Epidermal Growth Factor
ESPL1	Extra Spindle Pole Bodies Like 1
FANCA	Fanconi Anemia Complementation Group A
FC	Fold Change
FRMD5	FERM Domain Containing 5
GEO	Gene Expression Omnibus
ggplot2	Data Visualization Package in R
GO	Gene Ontology
GPIHBP1	Glycosylphosphatidylinositol Anchored High Density Lipoprotein Binding Protein 1
HFE	Hemochromatosis Protein
ID1	Inhibitor of DNA Binding 1
KEGG	Kyoto Encyclopedia of Genes and Genomes
KLF2	Kruppel Like Factor 2
KLF4	Kruppel Like Factor 4
LGI2	Leucine Rich Repeat LGI Family Member 2
LOXHD1	Lipoxygenase Homology Domains 1
MFAP3L	Microfibril Associated Protein 3 Like
miRecords	MicroRNA Records Database
miRNA	MicroRNA
miRTarBase	MicroRNA Target Database
MOK	MAPK/MAK/MRK Overlapping Kinase
mRNA	Messenger RNA
NCBI	National Center for Biotechnology Information
OSR2	Odd-Skipped Related Transcription Factor 2
PKA	Protein Kinase A
PPI	Protein–Protein Interaction
RASGRP2	RAS Guanyl Releasing Protein 2
RNA-seq	RNA Sequencing
RYR3	Ryanodine Receptor 3
SRA	Sequence Read Archive
TarBase	miRNA Target Database
TGF-β	Transforming Growth Factor-β
TIMP4	TIMP Metallopeptidase Inhibitor 4
TNFSF14	TNF Superfamily Member 14
TNS1	Tensin 1
TRAF4	TNF Receptor Associated Factor 4
