# Supplementary material for: Cross-Species Transcriptomic Integration Identifies a Conserved Molecular Core and Regulatory ceRNA Network in Duchenne Muscular Dystrophy
Source: Genes (Basel). 2026 Aug 27;17(9):1017. doi: 10.3390/genes17091017 (PMC13606584; doi:10.3390/genes17091017)
Supplement: Supplementary file 1 [file genes-17-01017-s001.zip › Supplementary Table S2.pdf]

Supplementary Table S2

Sensitivity analysis of cross-species differentially expressed gene (DEG) overlap using Benjamini–Hochberg false discovery rate (FDR)-adjusted p-values (padj < 0.05). Pairwise and three-way (Human/Mouse/Rat) overlaps of up- and down-regulated genes are shown for the *in vivo* comparisons. Under this stricter statistical threshold, the three-species concordant overlap was reduced to 7 genes (2 up-regulated, 5 down-regulated), compared with 62 concordant genes identified using the nominal p-value threshold ($p < 0.05$, $|log2FC| > 0.3$) applied as the primary screening criterion in this study (see Methods, Section 2.3).

Comparison	Up-Regulated Genes	Down-Regulated Genes
Human / Mouse	146	58
Mouse / Rat	5	12
Rat / Human	6	22
**Rat / Human / Mouse**	**2 (CCBE1, RFX2)**	**5 (CASKIN2, DMD, ID1, KLF2, KLF4)**
